# The value of the first postoperative diagnostic I-131 scan in patients with papillary thyroid carcinoma

**DOI:** 10.1007/s00432-023-05581-6

**Published:** 2024-02-06

**Authors:** Bingyu Ran, Jingjie Shang, Yong Chen, Miaoli Zhou, Huihu Li, Wenjun He, Yingxin Li, Qijun Cai, Bin Guo, Jian Gong, Hao Xu

**Affiliations:** Department of Nuclear Medicine, The First Affiliated Hospital of Jinan University, Guangzhou, Guangdong Province China

**Keywords:** Papillary thyroid carcinoma, Radioiodine scan, Diagnostic, Radioactive iodine

## Abstract

**Objective:**

To explore the feasibility of the postoperative diagnostic ^131^I whole-body planar scans (Dx-WBS) in papillary thyroid cancer (PTC) patients, and to clarify its value for accurate staging, risk stratification, and postoperative radioactive iodine (RAI) treatment management.

**Design:**

Retrospective study from 2015 to 2021.

**Setting:**

A total of 1294 PTC patients in the tertiary referral hospital.

**Participants:**

Patients with differentiated thyroid cancer who underwent total/subtotal thyroidectomy were included. Patients with non-PTC pathological type, non-first RAI treatment, and incomplete data such as Dx-WBS and postablation WBS (Rx-WBS) were excluded.

**Methods:**

The diagnostic efficacy of Dx-WBS was calculated with Rx-WBS as the reference. All patients were initially staged by the 8th edition of TNM staging, and risk stratification was performed based on clinical and pathological information. After Dx-WBS, the risk stratification was re-evaluated, and management was reconfirmed.

**Results:**

The detection rates of Dx-WBS for residual thyroid, cervical lymph nodes, upper mediastinal lymph nodes, lung, and bone distant metastasis were 97.6%, 78.3%, 82.1%, 66.7%, and 61.2%, respectively. The risk stratification of 113 patients (8.7%) changed after Dx-WBS, of which 107 patients changed from low to intermediate risk, 2 from low to high risk, and 4 from medium to high risk. A total of 241 patients (18.6%) adjusted the RAI regimen after Dx-WBS.

**Conclusion:**

This study confirms the diagnostic efficacy of the postoperative Dx-WBS in PTC patients and the value of Dx-WBS in accurately assessing risk stratification, as well as assisting in determining RAI treatment.

## Introduction

The incidence of differentiated thyroid cancers (DTC) continues to increase worldwide, almost entirely due to the increasing detection of papillary thyroid cancer (PTC) (Vaccarella et al. 2016). Most PTC are well differentiated, have relatively sedentary clinical behavior, and have mean 10-year survival rates between 80 and 95% (Sherman 2003; Jukic et al. 2022). Despite the long survival associated with PTC, approximately 20–30% of patients relapse over several decades, with two-thirds of these relapses occurring within the first decade after initial treatment (Brassard et al. 2011). Selective radioactive iodine (RAI) therapy plays a crucial role in minimizing the risk of tumor recurrence by treating possible residual tumors and regional/distant metastases (Ruel et al. 2015). An important goal of RAI therapy is to personalize treatment to maximize benefits.

To determine whether and how much RAI is given, the 2015 American Thyroid Association (ATA) guidelines developed a risk stratification system to manage patients with thyroid cancer recurrence or death, namely low, intermediate, and high risk mainly based on the primary tumor and regional/distant metastasis. They strongly recommend RAI for patients at high risk for recurrence, and consider it for those in the intermediate-risk category, but not routinely after thyroidectomy for low-risk PTC patients or those with single/multifocal microcancers (≤ 1 cm in diameter) (Haugen et al. 2016). Thus, adequate disease assessment, accurate TNM stage, and risk stratification are essential for formulating the appropriate treatment responses.

Neck ultrasonography (US) has a high sensitivity in detecting gross residual disease and cervical lymph node metastasis, but it is unable to detect micrometastasis and, occasionally, is limited in distinguishing postoperative changes and residual disease (Filetti et al. 2019). Small distant metastases may also be missed in computed tomography (CT). Undiagnosed regional and distant metastatic disease may contribute to subsequent clinical disease recurrence. In this context, more sensitive diagnostic tools are needed to identify diseases for which adequate RAI therapies are available. The postoperative diagnostic ^131^I whole-body planar scans (Dx-WBS) can improve the detection of occult functional locoregional disease and distant metastasis, and can also assess the range of the residual thyroid gland. However, its clinical value has been controversial. Supporters believe Dx-WBS diagnosis can clarify lesion status and guide RAI intake (Nostrand et al. 2009; Boom et al. 2021). Previous studies demonstrated that 25–53% of PTC patients’ clinical management could be altered based on the information provided by Dx-WBS. (Nostrand et al. 2009; Avram et al. 2013; Chen et al. 2012). The opposing side holds that Dx-WBS provides limited clinical information, which cannot change the clinical management plan, has no effect on the long-term prognosis of patients (Schlumberger and Pacini 2009; Salvatori et al. 2004), and may also produce a "stunning effect". Therefore, the aims of this study are to clarify the efficacy of Dx-WBS in detecting lesions based on a large sample in our center, further explore the effect of Dx-WBS on the staging and risk stratification of PTC patients, and evaluate its value in determining RAI treatment.

## Method

### Patients

From January 2015 to December 2021, we retrospectively reviewed 1895 postoperative patients with histologically confirmed DTC scheduled for RAI treatment in our center. All patients restricted dietary iodine intake for 2 weeks and were prepared for thyroid hormone withdrawal for at least 4 weeks or did not start thyroid hormone replacement therapy after thyroid cancer surgery. Patients were excluded if they were PTC, with no available data of Dx-WBS examination, with no complete clinical data, and had received previous RAI treatment. This study was approved by the Research Ethics Committee of the First Affiliated Hospital of Jinan University and complied with national legislation and the guidelines of the Declaration of Helsinki. Due to the retrospective nature of this study, informed consent was not required.

### Image acquisition and analysis

A GE Optima NM/CT640 multi-function single-photon emission computed tomography/computed tomography (SPECT/CT) with a high-energy collimator were used. Planar scintigraphy was performed 1 day after ingestion of 74 MBq (2 mCi) of ^131^I. Whole-body and static neck images were acquired in anterior and posterior projections. The patients who showed radioiodine avidity unclearly or outside the thyroid bed also received additional SPECT/CT to definite and localize the lesion. Patients in this study were treated with doses of 100 to 200 mCi, and postablation WBS (Rx-WBS) was administered 3–4 days after administration.

Two experienced nuclear medicine physicians unblinded to the patient’s clinical and biochemical information and pathology reports interpreted the image information. When their diagnoses were discordant, a third physician reviewed the images. Regional radioiodine uptake in the whole body was visually classified into four categories (Avram et al. 2013): (1) neck uptake lesions located within the thyroid bed, which are suggestive of residual thyroid tissue; (2) neck uptake focus outside the thyroid bed and uptake lesions in upper mediastinum, which likely represent lymph node metastasis; (3) abnormal uptake lesions located in the axillary, lung, bone, etc.., which indicate distant metastasis; (4) no abnormal uptake in the whole body. Radioiodine avid lesions identified in the Dx-WBS and the Rx-WBS were compared qualitatively by visual assessment.

### Clinical impact evaluation

According to the staging system of the 8th edition of the American Joint Committee on Cancer (AJCC)/TNM Cancer Staging (Tuttle et al. 2017) and the 2015 ATA Guidelines (Haugen et al. 2016), the pre-Dx-WBS stage and risk stratification for recurrence were determined based on the pathological data and conventional imaging examinations, such as US, CT, and bone scintigraphy. Integrated Dx-WBS results with conventional examinations, the post-Dx-WBS stage and risk stratification were then determined based on the same guidelines. Changes in the TNM stage and recurrence risk stratification were recorded for all patients.

### Management plan

From electronic medical records, both the intended management plan before WBS and the actual management plan after WBS were recorded. The therapeutic dose of RAI was determined according to the 2015 ATA guidelines (Haugen et al. 2016), which generally divided into three gradients: patients with PTC in the low-risk state were usually given 3.70 GBq (100 mCi), the intermediate-risk stage were given a second gradient dose of 3.70–5.55 GBq (100–150 mCi), and the high-risk state were given a third gradient dose of 5.55–7.40 GBq (125–200 mCi). The RAI dose of children (age < 18 years) was 1/2–5/6 of that of adults (Jarzab et al. 2005). The individual dose for each patient is determined comprehensively based on clinical information (including tumor size, invasion and other tumor characteristics, postoperative residual thyroid tissue, thyroid function indicators, health status, etc.).

### Statistical analysis

All data were presented as number (percentage) for categorical variables, median (range), and mean ± SD for continuous variables. The McNemar test was used to determine whether there was a significant association between any two of the imaging results, and *p* < 0.05 was considered significant. All data were processed with SPSS software (version 26.0).

## Results

### Clinical characteristics

**Fig. 1:**
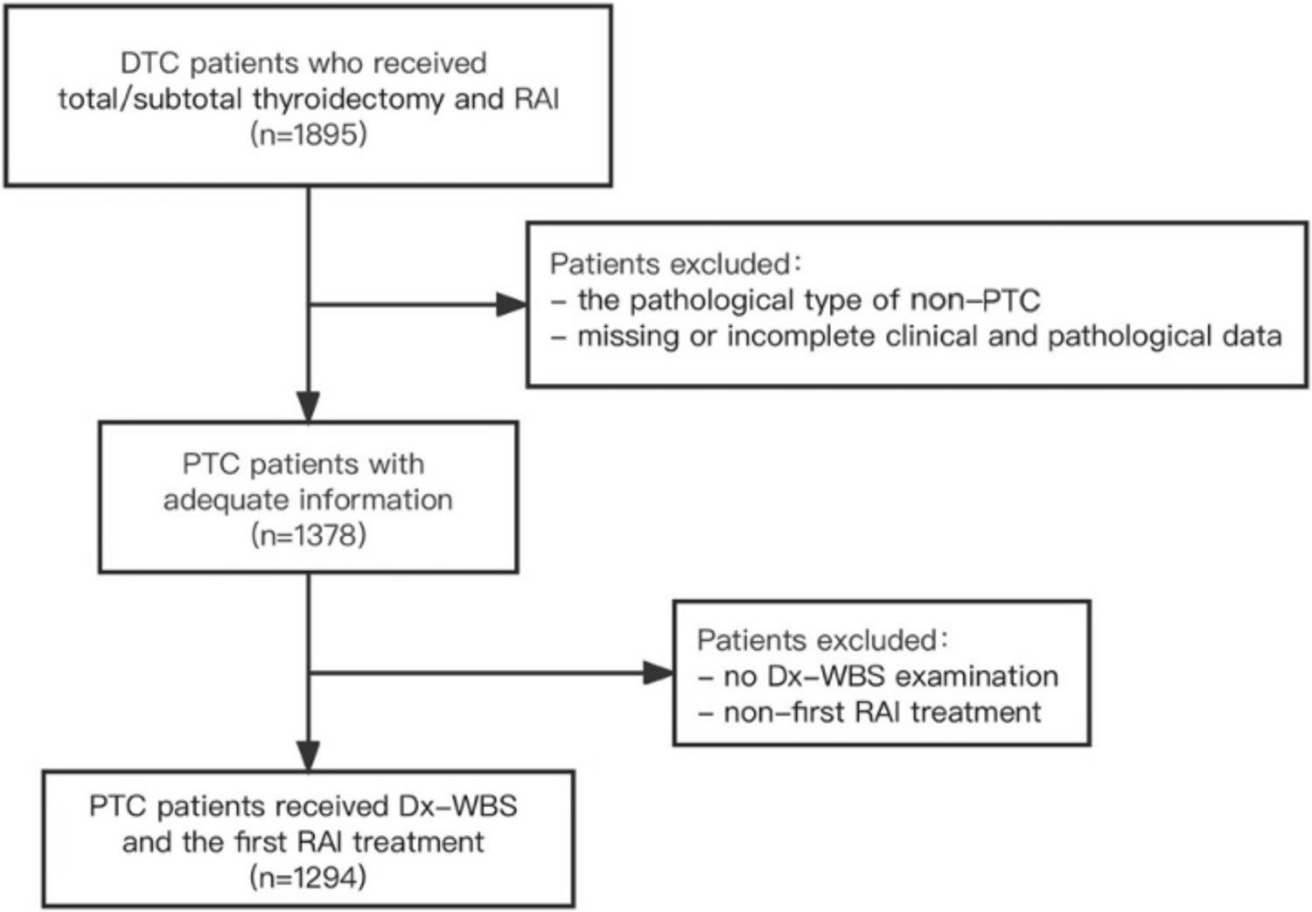
Study screening flow chart. DTC, differentiated thyroid cancers; RAI, radioactive iodine; PTC, papillary thyroid cancer; Dx-WBS, diagnostic 131I whole-body scan

A total of 1294 patients were enrolled in the study, including 921 females (71.2%) and 373 males (28.8%). The mean age of all patients was 39.5 ± 12.4 years (range 12–80 years). A flow diagram of the study is shown in Fig. 1.

Among them, thyroglobulin antibody positive accounted for 8.6% and stimulated thyroglobulin levels (ps-Tg) greater than or equal to 1.0 ng/ml accounted for 62.9%. In the initial postoperative stage, 197 cases (15.2%) had no definite regional lymph node metastasis, and 1257 cases (97.1%) were not found to have distant metastasis. All patients underwent chest CT before Dx-WBS, and 37 had positive findings. Among them, 34 had lung metastasis only, 1 had bone metastasis only, and 2 had both lung and bone metastasis. Ten patients had whole-body bone scintigraphy before Dx-WBS, and in one of them an uptake focus was found in the frontal bone. In this study, 15 children (1.2%, 12–18 years) were included. Table 1 shows the baseline characteristics of the respondents.

**Table 1:** the baseline characteristics of the patients

Characteristics	Number of patients (%)
*Age*	
< 55 years	1105 (85.39)
≥ *55 years*	
Sex	189 (14.61)
Male	373 (28.83)
Female	921 (71.17)
PTMC	735 (56.80)
*Pathological lymph node metastasis*	
Absence	197 (15.22)
Presence	1097 (84.78)
*Initial distant metastasis*	
Absence	1257 (97.14)
Presence	37 (02.86)
*Thyroid stimulating hormone*	
< 30 mIU/L	169 (13.06)
≥ 30 mIU/L	1125 (86.94)
*Anti-thyroglobulin antibodies*	
Negative	1183 (91.42)
Positive	111 (08.58)
*Stimulated thyroglobulin levels*	
< 1 ng/ml	480 (37.09)
1 to < 10 ng/ml	493 (38.10)
10 to < 30 ng/ml	159 (12.29)
≥ 30 ng/ml	162 (12.52)
Free thyroxine (pmol/L)	3.2 (0–18.86)^a^

*PTMC* papillary microcarcinoma

^a^presented as median (range)

### Diagnostic efficacy of Dx-WBS

In our study, 1222 of 1294 patients (94.4%) patients showed remnant thyroid activity on Dx-WBS; 262 patients (20.4%) exhibited regional nodal metastases, and 36 (2.8%, including 30 bone metastases and 4 lung metastases) had distant metastases. Rx-WBS was repeated after RAI and showed residual thyroid uptake in 1252 (96.8%) patients, 309 (23.9%) for regional lymph node metastases, and 58 (4.5%, including 49 bone metastases and 6 lung metastases) for distant metastases. One patient had abnormal uptake foci above the bladder in both Dx-WBS and Rx-WBS and was finally confirmed to be a teratoma by pathology.

The distribution of radioiodine foci on Dx-WBS was compared with subsequent Rx-WBS (Table 2). In 1182 of 1294 patients (81.5%), the imaging findings on Dx-WBS and Rx-WBS were concordant. In contrast to Rx-WBS, Dx-WBS has a positive detection rate of up to 97.6% in the residual thyroid, followed by 82.1% in the superior mediastinum lymph nodes, 78.3% in cervical lymph nodes, 66.7% in bone metastases, and 61.2%in lung distant metastases. There was no significant difference between Dx-WBS and Rx-WBS in identifying the superior mediastinum lymph nodes (p = 0.40) and bone metastases (p = 0.53) in all PTC patients. However, Dx-WBS still has limitations in diagnosing residual thyroid, cervical lymph nodes, and lung metastases needs to be considered. (*p* < 0.05).

**Table 2:** Findings and diagnosis rate of preablation Dx-WBS in different groups

	Thyroid residual	Cervical lymph nodes	Superior mediastinum lymph nodes	Bone metastases	Lung metastases
All patients	97.6 (1222/1252)^*^	78.3 (242/309)*	82.1 (32/39)	61.2 (30/49)*	66.7 (4/6)
Age < 55 years	97.7 (1049/1074)	78.5 (208/265)*	82.4 (28/34)	61.0 (25/41)*	75.0 (3/4)
Age ≥ 55 years	97.3 (173/178)	77.3 (34/44)	80.0 (4/5)	62.5 (5/8)	50.0 (1/2)
PTMC	96.8 (358/370)	82.1 (69/84)	85.7 (6/7)	66.7 (4/6)	100.0 (2/2)
Male	98.1 (361/368)	81.0 (94/116)	86.7 (13/15)	53.8 (7/13)	0 (0/1)*
Female	97.4 (861/884)	76.7 (148/193)*	79.1 (19/24)	63.9 (23/36)	80.0 (4/5)
ps-Tg < 10 ng/ml	97.0 (795/820)*	79.2 (141/178)*	70.6 (12/17)	33.3 (3/9)	100 (3/3)
ps-Tg ≥ 10 ng/ml	99.7 (308/309)	76.0 (76/100) *	93.3 (14/15)	65.8 (25/38)	33.3 (1/3)

Data in parentheses are the raw data used to calculate percentages. *p* values were calculated with the chi-square test to compare Dx-WBS and Rx-WBS in terms of detected lesions

*Dx-WBS*, postoperative diagnostic 131-radioiodine whole-body scan; *PTMC*, papillary microcarcinoma

*The* p* values of paired the McNemar test were less than 0.05

Further subgroup analysis showed that in patients aged 55 years or older, no significant differences were found between the two images in residual thyroid, superior mediastinal lymph node metastasis, and distant metastasis (all of *p* > 0.05). In patients younger than 55 years, no significant differences were found in residual thyroid, superior mediastinal lymph node metastasis, and bone metastases (*p* > 0.05). In addition, in patients with papillary thyroid microcarcinoma (PTMC), there were no significant differences between Dx-WBS) and Rx-WBS in detecting residual thyroid tissue (p=0.60), cervical/upper mediastinal lymph nodes (p=0.21, p=0.78), as well as pulmonary (p=0.53) and bone distant metastases (p > 0.99). In patients negative for thyroglobulin antibody (TgAb), regardless of pre-stimulated thyroglobulin (ps-Tg) levels, the diagnostic efficacy of Dx-WBS was not significantly different from (Rx-WBS for upper mediastinal, pulmonary, and bone metastases. In male patients, Dx-WBS showed inferior diagnostic ability for bone metastases compared to Rx-WBS (p = 0.01).

### Impact of Dx-WBS on clinical assessment

The pre- and post-Dx-WBS stage and risk stratification for all the patients and subgroups are shown in Table 3. For all of the patients’ pre-Dx-WBS stage, 1102 patients (85.2%) had stage I disease, 148 (11.4%) stage II, 35 (2.7%) stage III, and 9 (0.7%) stage IV. For the post-Dx-WBS stage, 1089 patients (84.2%) had stage I disease, 160 (12.4%) stage II, 34 (2.6%) stage III, and 11 (0.9%) stage IV. Overall, 15 patients (1.2%) were re-staged following Dx-WBS (Table 4). Nine patients (< 55 y) were upstaged from stage I to stage II based on finding radioiodine-avid distant disease. Four patients (≥ 55 y) were upstaged from stage I to stage II based on finding radioiodine-avid disease in regional lymph nodes. Furthermore, because distant metastatic disease was detected, two patients (≥ 55 y) were upstaged from stage II to stage IV and from stage III to stage IV, respectively.

**Table 3:** The pre- and post-Dx-WBS stage and risk stratification for all the patients and subgroups

	Patient No				Patient No		
	I	II	III	IV	Low risk	Intermediate risk	High risk
*All patients*							
Pre-Dx-WBS	1102	148	35	9	280	782	232
Post-Dx-WBS	1089	160	34	11	171	885	238
*Age* < *55 years*							
Pre-Dx-WBS	1077	28	/	/	232	187	686
Post-Dx-WBS	1068	37	/	/	139	192	774
*Age* ≥ *55 years*							
Pre-Dx-WBS	25	120	35	9	48	45	96
Post-Dx-WBS	21	123	34	11	32	46	111
*PTMC*							
Pre-Dx-WBS	335	39	/	2	126	245	5
Post-Dx-WBS	332	42	/	2	82	288	6
*Male*							
Pre-Dx-WBS	324	39	6	4	73	231	69
Post-Dx-WBS	319	44	6	4	38	265	70
*Female*							
Pre-Dx-WBS	778	109	29	5	207	551	163
Post-Dx-WBS	770	116	28	7	133	620	168
*ps-Tg* < *10 ng/ml*							
Pre-Dx-WBS	592	65	19	0	175	401	100
Post-Dx-WBS	588	69	18	1	117	456	103
*ps-Tg* ≥ *10 ng/ml*							
Pre-Dx-WBS	235	52	10	8	37	184	84
Post-Dx-WBS	229	58	10	8	15	204	86

*Dx-WBS*, postoperative diagnostic 131-radioiodine whole-body scan; *PTMC*, papillary microcarcinoma

**Table 4:** Changes in TNM stage and risk stratification for disease recurrence with Dx-WBS results

	Patient no. (%*)			Patient no. (%*)		
	I–II	II–IV	III–IV	Low to intermediate	Low to high	Intermediate to high
Total	13 (1.2)	1 (0.7)	1 (2.9)	107 (38.2)	2 (0.7)	4 (0.5)
< 55 y	9 (0.8)	0	0	92 (39.7)	1 (0.4)	4 (0.6)
≥ 55 y	4 (16.0)	1 (0.8)	1 (2.9)	15 (31.3)	1 (2.1)	0
PTMC	3 (0.9)	0	0	44 (34.9)	0	1 (0.4)
Male	5 (1.5)	0	0	35 (47.9)	0	1 (0.4)
Female	8 (1.0)	1 (0.9)	1 (3.4)	72 (34.8)	2 (1.0)	3 (0.5)
ps-Tg < 10 ng/ml	4 (0.7)	0	1 (5.3)	58 (33.1)	0	3 (0.7)
ps-Tg ≥ 10 ng/ml	6 (2.6)	0	0	21 (56.8)	1 (2.7)	1 (0.5)

*Dx-WBS*, diagnostic ^131^I whole-body scan; *PTMC*, papillary microcarcinoma

*Percentage of the change, the number of changes/the original number of this group

For all of the patients, the pre-Dx-WBS risk stratification for recurrence was low-risk in 280 patients (21.6%), intermediate-risk in 782 (60.4%), and high-risk in 232 patients (17.9%). According to the Dx-WBS results, 171 patients (13.2%) had low-risk, 885 (68.4%) had intermediate-risk, and 238 (18.4%) had high-risk subjects were finally assigned. Overall, the risk stratification of 113 patients (8.7%) was upgraded (Table 4). Of these 133 patients, Dx-WBS upstaged 107 patients from low-risk to intermediate-risk by detecting nodal metastatic lesions, and upstaged 6 patients from low- or intermediate-risk to high-risk by detecting distant metastases. Furthermore, Dx-WBS upstaged 44 and 1 patients with PTCM from low-risk to intermediate-risk and from intermediate-risk to high-risk, respectively. The total changes of Dx-WBS in grading and recurrence risk stratification of males (1.3% and 9.7%) were higher than those of female patients (1.1% and 8.4%). Among 981 TgAb negative PTC patients, Dx-WBS changed the stage of 1.6% and recurrence risk stratification of 20% in patients with ps-Tg < 10 ng/ml (305 cases). In patients with ps-Tg ≥ 10 ng/ml (676 cases), 1.0% stage and 4.0% risk were changed.

### Impact of Dx-WBS on management plan

**Fig. 2:**
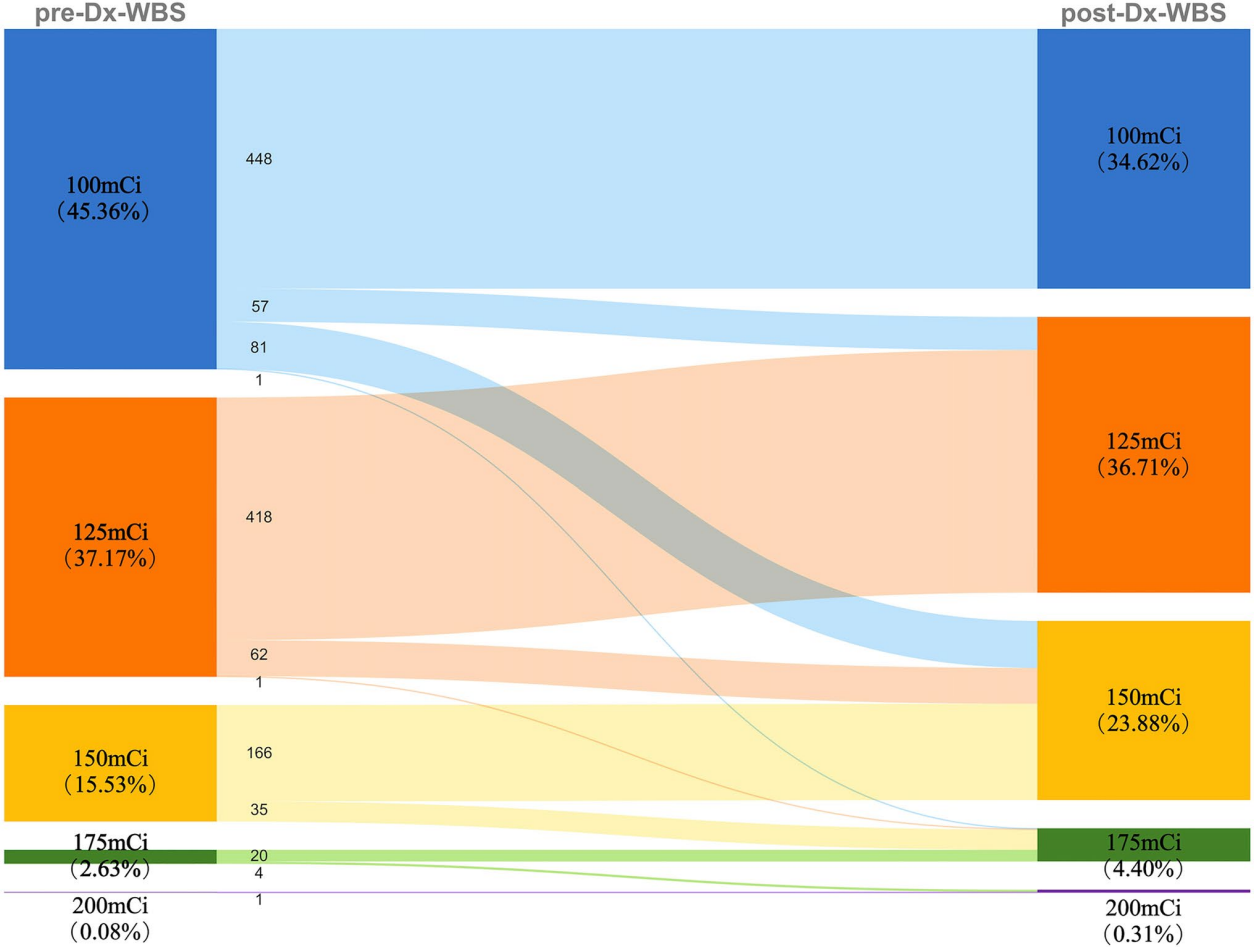
The changes in the absorbed dose of the patients before and after Dx-WBS. Dx-WBS, diagnostic 131I whole-body scan

The therapeutic management was altered following a Dx-WBS scan in 241 patients (18.6%). Figure 2 shows in detail the changes in the absorbed dose of the patients.

In patients with the original plan to take a dose of 100 mCi orally, due to the Dx-WBS regional lymph node metastases, 57 patients’ RAI dose increased to 125 mCi, 79 increased to 150 mCi; three additional patients were upgraded to 150 mCi and 175 mCi, respectively, because of the diagnosis of distant metastases.

For patients with initial doses in the 125 mCi setting, 71 patients were found to have regional lymph node metastases on Dx-WBS which caused 59 patients to change doses to 150 mCi, with the remaining 12 patients still retaining 125 mCi, depending on individual characteristics; Four patients had distant metastases, of whom three received 150 mCi and one got 175 mCi.

Among patients who were initially given 150 mCi, 33 had cervical/upper mediastinal lymph node metastases after Dx-WBS, 10 increased from 150 to 175 mCi, and 23 remained on 150 mCi. Another 25 PTC patients who should also have taken 150 mCi were upgraded to 175 mCi because they showed distant metastases.

In four patients with an initial RAI dose of 175 mCi, Dx-WBS identified lung metastases and 200 mCi was eventually given.

In the group of 15 pediatric patients, 5 cases (33.3%) underwent a change in their treatment dosage following Dx-WBS. Four patients had their RAI treatment dosage adjusted due to regional lymph node metastases coupled with higher ps-Tg levels: two cases increased from 100 to 125 mCi, and the other two from 125 to 150 mCi. One child's dosage was elevated from 125 to 150 mCi due to bilateral pulmonary metastases. In Addition, although distant metastases were detected in 3 patients on Dx-WBS, their treatment dosage was still maintained at 150 mCi.

## Discussion

In this study, Dx-WBS and Rx-WBS were compared and patients' TNM stage and risk stratification were reassessed after Dx-WBS. Simultaneously, we retrospectively analyzed the dosage changes in RAI treatment before and after Dx-WBS in PTC patients. Eventually, we confirmed the diagnostic efficacy of Dx-WBS and provided clinical evidence that Dx-WBS can provide a more precise assessment of patients' TNM staging and risk stratification because of its ability to accurately identify positive cases, therefore assisting in the provision of more appropriate personalized treatment management for patients.

Our research demonstrates that Dx-WBS has a positive detection rate of over 75% for residual thyroid and regional lymph node metastases and a positive rate of over 60% for distant metastases in the bones and lungs. Avram A M et al., in a study of 303 patients with DTC, showed that 6% had additional foci on Rx-WBS; Of these, only 1.4% of post-treatment scans showed metastatic disease (Avram et al. 2013). Similar to them, Danilovic DLS et al. found that compared with Rx-WBS, Dx-WBS had a detection rate of about 76% for cervical lymph nodes, and 80% and 70% for bone and lung metastases, respectively (Danilovic et al. 2022). Our results show a lower positive rate of lesion positivity with Dx-WBS, possibly because we studied only planar imaging and did not combine SPECT/CT findings. Our centers traditionally performed planar imaging before RAI, adding SPECT/CT only when equivocal lesions or metastases were suspected. This can shorten the examination time and reduce the financial burden for patients. There might also be a possibility that the larger sample size led to more false negatives. Overall, the potential of Dx-WBS as a diagnostic tool is identifying the presence of pre-thyroidectomy RAI as well as the detection of metastases in both regional and distant areas. In addition, we further analyzed the statistical difference in the detection rate of Dx-WBS versus Rx-WBS. The results showed that Dx-WBS showed better diagnostic efficacy in PTMC and elderly patients. In the two groups of patients, Dx-WBS had the same diagnostic efficacy as Rx-WBS in the detection of residual thyroid, cervical/mediastinal lymph nodes, bone and lung metastasis detection (*p* ≥ 0.05).

Dx-WBS revealed probable regional metastases in 20.3% (262/1294) of patients and distant metastatic disease in 2.8% (36/1294) of all patients, changing TNM staging in 0.8% (9/1105) younger patients (age < 55 years) and 3.2% (6/189) older patients (age ≥ 55 years), as well as risk stratification in 8.7% (113/1294) of all patients. The change in risk stratification as compared with stage after Dx-WBS is more pronounced because the vast majority (85.4%) of patients in our study were young patients in whom unexpected regional lymph node metastases did not alter stage.

In addition to its impact on disease hierarchy, the utility of diagnostic imaging research depends more importantly on its impact on management. Although the identification of partial residual thyroid and regional metastases, or even distant metastases, does not alter staging or risk stratification, it may affect management decisions. Incorporating Dx-WBS into clinical decision-making algorithms for thyroid cancer can advance the personalization of treatment strategies. In two prospective studies of 320 patients conducted by Avram et al. (2013, 2015), it was found that Dx-WBS combined with SPECT/CT changed the staging in 4% of young patients and 25% of elderly patients, the risk of recurrence in 15%, and the clinical management in 29.4% of patients changed. Similar to the above results, Wong et al. (2010) also used WBS combined with SPECT/CT to perform pre-RAI scans on 48 DTC patients, and the results changed the staging of 21% of the patients and increased the RAI treatment dose of 29%. Van Nostrand et al. (2009) showed that Dx-WBS provided information that could alter clinical management in 53% of patients. In the present study, the RAI treatment dose was changed in 18.6% of patients based on the Dx-WBS results. One hundred and seventy-one Dx-WBS negative low-risk patients with PTC, who are not recommended for RAI treatment according to the guidelines, but due to a strong appetite for patient autonomy, were eventually also treated with ancillary therapy.​In addition, one of our exclusion criteria was patients who were not treated with RAI, so referral surgery and delayed RAI were not included in the changed treatment regimen. Similar to the results of Kaewchur et al. (2021), we also conducted a subgroup analysis of thyroid microcarcinoma. The results showed that Dx-WBS changed the risk stratification of 12.0% PTMC and the management plan of 18.9%, which is higher than the change rate of PTC as a whole. This seems to indicate that Dx-WBS is of greater clinical value for disease assessment and treatment decision-making in PTMC patients.

Undiagnosed regional and distant metastatic disease can lead to subsequent clinical disease recurrence. Moreover, according to ATA recommendations, ps-Tg measurement is a crucial tool in the care of PTC patients. Several studies have reported the use of ps-Tg values to exclude the risk of metastasis, consequently ruling out the possibility of TRA (Rosario et al. 2011; Rosario and Purisch 2008). However, in routine clinical monitoring, there are a large number of patients who have radioiodine-heated metastatic lesions on imaging following RAI therapy but have negative TgAb and undetectable or low serum ps-Tg values (< 1 ng/ml) (Campenni et al. 2018; Robenshtok et al. 2013; Giovanella et al. 2011). Our study showed that in patients with ps-Tg < 10 ng/ml, Dx-WBS altered the staging in 1.6% and the recurrence risk stratification in a substantial 20% of the patients. This suggests that Dx-WBS has particular potential in identifying subclinical disease in patients with relatively lower ps-Tg levels, where other diagnostic modalities might be less informative. Dx-WBS provides valuable information for disease localization and detection, which complements histopathology, thyroglobulin, and traditional imaging information, to the comprehensive assessment of disease with more accurate staging and risk stratification, guide treatment decisions, and monitoring intensity.

Although our research results indicate differences in lesion detection between diagnostic whole-body scintigraphy (Dx-WBS) and post-treatment whole-body scintigraphy (Rx-WBS), it cannot be denied that a certain number of patients with iodine-avid lesions are still detected after Dx-WBS examination. This finding provides clinicians with a more comprehensive understanding of the patients' true disease status, facilitating the development of more personalized treatment plans.

Our study has several limitations. First, we did not study planar imaging with SPECT/CT because patients at our center routinely undergo planar imaging before RAI and SPECT/CT is performed only when the lesion is ambiguous. Second, despite the large amount of data in our center, it is a retrospective study, and prospective studies need to be designed for further confirmation. In addition, some selection bias may occur due to the single-center study.

In conclusion, Dx-WBS can effectively identify the residual disease, regional lymph nodes, and distant metastasis of PTC patients after surgery, assess the disease status more fully, change the TNM classification and risk stratification of patients, and ultimately assist in ensuring that patients receive the appropriate level of intervention. Dx-WBS before RAI has a certain clinical value for PTC patients.

## Data Availability

The datasets generated during and/or analysed during the current study are available from the corresponding author on reasonable request.
